# Simultaneous trimodal MR-PET-EEG imaging for the investigation of resting state networks in humans

**DOI:** 10.1186/2197-7364-2-S1-A71

**Published:** 2015-05-18

**Authors:** Irene Neuner, Joerg Mauler, Jorge Arrubla, Elena Rota Kops, Lutz Tellmann, Jurgen Scheins, Hans Herzog, Karl Josef Langen, Jon Shah

**Affiliations:** RWTH Aachen, Germany; Institute of Neuroscience and Medicine – 4, Forschungszentrum Juelich GmbH, Germany

Glucose is the principal source of energy for the brain and its relationship to neuronal activity are poorly understood. The human brain uses 80% of its energy for ongoing neural activity that occurs in isolation from any particular stimulus. A promising tool for the investigation of glucose metabolism and its relationship to neuronal activity is simultaneous trimodal MR-PET-EEG data imaging. We here demonstrate the first in vivo human trimodal data at 3T. In one session MR, FDG-PET and EEG data were recorded simultaneously at a 3T hybrid MR-BrainPET scanner (Siemens, Germany) equipped with a 32 channel MR-compatible EEG system (Brain Products, Germany) in 11 healthy volunteers (11 males, mean age: 25.2 years SD: 1.2). MR and EEG data acquisition MP-RAGE (TR = 2250 ms, TE= 3.03 ms, 176 sagittal slices. 1 mm, GRAPPA factor 2. MR-based attenuation correction of PET data via UTE: flip angle=15. Two different echo times TE1=0.07 and TE2=2.46 ms, TR=200 ms. EPI sequence (TR: 2.2 s, TE: 30 ms, FOV: 200 mm, 165 volumes, The subjects were requested to close their eyes and relax EEG data were recorded using a 32-channel MR compatible EEG system. App. 200 MBq/μmol FDG were injected, data were acquired in list mode and iteratively reconstructed with all necessary corrections into 153 slices with 256 x 256 voxels sized 1.25 mm^3^. The trimodal approach, recording PET data, MR data and EEG data simultaneously was successful. The high neuronal activity of the structures within the default mode network occurs on the basis of a high glucose consumption rate within the default node network. The activity of the default mode is not tied to a special EEG frequency band.

